# P-310. Patient-reported Outcomes of Long-Acting Injectable Cabotegravir Versus Oral PrEP Use for HIV Prevention: Sub-analysis of the AURORA study

**DOI:** 10.1093/ofid/ofaf695.529

**Published:** 2026-01-11

**Authors:** W David D Hardy, Angela D Settle, Kelly E Pillinger, Leah Molloy, Laura Simone, Chris Napolitan, Chelsie Chadha, Melissa Rodriguez, Jeffrey D Carter

**Affiliations:** Keck School of Medicine of the University of Southern California, Los Angeles, California; West Virginia Health Right, Inc., Charleston, West Virginia; PRIME Education, New York, NY; PRIME Education, New York, NY; PRIME Education, LLC, Fort Lauderdale, Florida; PRIME Education, New York, NY; PRIME Education, New York, NY; PRIME Education, New York, NY; PRIME Education, LLC, Fort Lauderdale, Florida

## Abstract

**Background:**

Long-acting injectable cabotegravir (CAB-LA) has been FDA-approved since 2021, yet real-world perspectives of CAB-LA PrEP users’ overall satisfaction, acceptance and stigma related to its’ use remain limited, especially in comparison to oral PrEP users.

**Figure:**
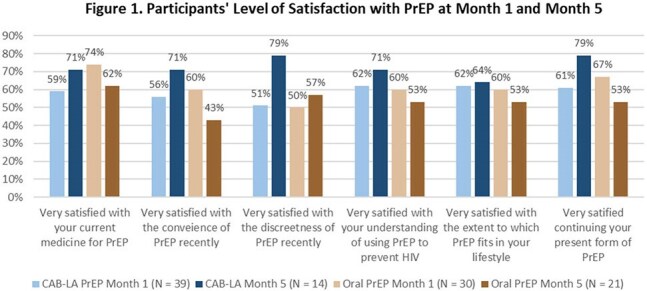
Participants' Level of Satisfaction with PrEP at Month 1 and Month 5

**Figure:**
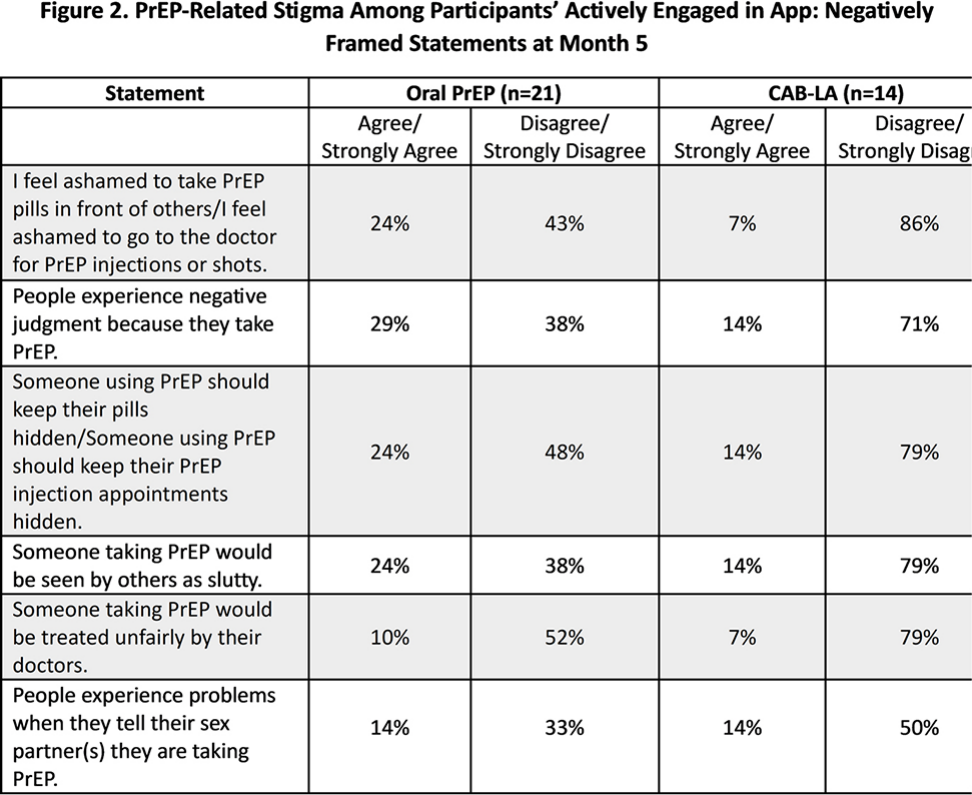
PrEP-Related Stigma Among Participants’ Actively Engaged in App: Negatively Framed Statements at Month 5

**Methods:**

Participants (≥ 18 years old) prescribed CAB-LA, daily FTC/TDF, or daily FTC/TAF for HIV prevention by providers at West Virginia Health Right in Charleston, WV and with access to a smartphone were eligible for enrollment (October 2023-November 2024) in the 1-year study. Participants received access to a customized digital health app through Reciprocity Health® that surveyed patient-reported outcomes (PROs), including medication satisfaction, acceptance, and PrEP-related stigma. Modest financial incentives encouraged users to reach app milestones. Interim analysis of PROs through month 5 were analyzed using descriptives statistics and chi-square tests.

**Results:**

There were 123 participants enrolled; 76 actively engaged in the app to date (N=45 CAB-LA; N = 31 oral PrEP) and completed PRO surveys. Overall, the proportion of PrEP users who reported being very satisfied across 6 measures of satisfaction increased in the CAB-LA group from month 1 to month 5 (mean: 59% vs 73%, p = .41), while it slightly declined in the oral PrEP group (mean: 62% vs. 54%, p = .44) (Fig 1). Among users still on PrEP, the percentage reporting challenges fluctuated among CAB-LA users (16% at month 1, 18% at month 3, and 10% at month 5) and oral PrEP users (23%, 43%, and 16%, respectively). At month 3, the main challenge for CAB-LA users was keeping up with visits/labs (6%), while oral PrEP users struggled with adherence (26%), visits/labs (13%), and transportation (13%). Stigma-related PROs showed 29% of oral PrEP users agreed that people experience negative judgement due to taking PrEP vs. 14% of CAB-LA users (Fig 2). Additionally, 67% of oral PrEP users and 93% of CAB-LA users felt proud to take PrEP.

**Conclusion:**

Overall PrEP satisfaction among CAB-LA users increased over time and was numerically higher than oral PrEP users at month 5. Additionally, oral PrEP users experienced greater levels of stigma compared to CAB-LA users.

**Disclosures:**

W David D. Hardy, MD, Gilead Sciences: Advisor/Consultant|Merck: Advisor/Consultant|ViiV Healthcare: Advisor/Consultant

